# *Helicobacter pylori* Recurrence after Eradication Therapy in Jiangjin District, Chongqing, China

**DOI:** 10.1155/2020/7510872

**Published:** 2020-04-06

**Authors:** Gang Zhou

**Affiliations:** Department of Gastroenterology, Central Hospital of Jiangjin District, Chongqing 402260, China

## Abstract

**Purpose:**

To investigate the recurrence rate of *Helicobacter pylori* infection after eradication in Jiangjin District, Chongqing, China, and to analyze the related causes.

**Methods:**

Outpatients who were eradicated of *H. pylori* infection with standard therapy between August 2014 and August 2017 were included in this study. The recurrence rate was investigated 1 year later. Data regarding gender, smoking, alcohol intake, frequency of eating out, and treatment strategy were recorded, and their relationships with the recurrence rate were analyzed. Multivariate logistic regression analysis was performed to determine the independent risk factors for *H. pylori* infection recurrence.

**Results:**

In total, 400 patients (225 males and 175 females) were included in this study. Of them, the recurrence rate of *H. pylori* infection was 4.75% (19/400), with 5.33% (12/225) in males and 4.57% (7/175) in females, showing no gender difference. The recurrence rate was 7.03% (9/128) in smokers and 3.68% (10/272) in nonsmokers, while it was 6.45% (12/186) in those who drink alcohol and 3.27% (7/214) in those who do not drink alcohol, showing no significant differences. The higher the frequency of eating out, the higher the recurrence rate of *H. pylori* infection (*P* = 0.001). There was a statistically significant difference in the recurrence rate between patients receiving treatment alone and patients whose family members also received treatment (6.08% vs. 0.96%, *P* = 0.035). Drinking and dining out were independent risk factors for *H. pylori* infection recurrence (*P* = 0.014 for drinkers and *P* = 0.015 and *P* = 0.003 for those who sometimes and often dine out, respectively).

**Conclusions:**

The overall recurrence rate after *H. pylori* eradication by standard therapy in Jiangjin District is 4.75%. Reducing the frequency of eating out and family members receiving treatment may reduce the recurrence of *H. pylori* infection.

## 1. Introduction

Chronic gastritis is one of the most common life-long inflammatory diseases. More than half of the world's population are estimated to have chronic gastritis to some extent [1]. *Helicobacter pylori* infection is one major cause of chronic gastritis. About 20% of *H. pylori*-infected patients develop peptic ulcers, and 1% of infected patients develop gastric malignancies, including gastric cancer and mucosa-associated lymphoid tissue lymphoma [2–4]. In addition, *H. pylori* is thought to be associated with some extragastric disorders, such as cardiovascular, skin, and blood system diseases [3, 4].

The prevalence of *H. pylori* infection varies greatly geographically. In developing countries, it is estimated that more than 80% of the population is *H. pylori* positive, even in children and adolescents, while in developed countries, less than 40% of the population is *H. pylori* positive, and children have a lower rate of infection than adults and the elderly [5]. Since *H. pylori* infection is very common and leads to many diseases, both domestic and international guidelines recommend eradication therapy for *H. pylori*-infected patients [6–8]. However, studies have shown that despite regular treatment, there is still a risk of *H. pylori* recurrence [9–11], with a higher rate in developing countries than in developed countries. *H. pylori* recurrence is defined as negative detection of *H. pylori* at 4 weeks after eradication therapy but positive detection at some later time [12]. *H. pylori* recurrence can occur either by recrudescence or reinfection. Recrudescence refers to the recolonization of the same strain, while reinfection refers to colonization with a new strain [9, 10]. Most cases of *H. pylori* recurrence are due to recrudescence.

Many risk factors for *H. pylori* infection have been reported, including socioeconomic factors, education, family density, lifestyle, and other factors [13–16]. These factors are also possible risk factors for *H. pylori* recurrence, since reinfection is one form of recurrence. A meta-analysis has shown that *H. pylori* recurrence rates are significantly and inversely correlated with socioeconomic development metrics [17].

*H. pylori* recurrence after eradication will reduce the clinical significance of eradication of *H. pylori*, inevitably increase the difficulty of drug selection, and aggravate *H. pylori* resistance [9, 18]. The *H. pylori* infection rate is 54.59% in western Chongqing [19], which is a high-prevalence area with many patients, but the recurrence rate of *H. pylori* infection remains unclear. This study was aimed at investigating the recurrence rate of *H. pylori* infection after eradication in patients living in Jiangjin District, Chongqing, China, and at examining the related factors.

## 2. Materials and Methods

### 2.1. Patients

From August 2014 to August 2017, outpatients with *H. pylori* infection confirmed by the ^14^C-urea breath test from Jiangjin District, Chongqing, China, were enrolled in this study. The outpatients received a quadruple treatment regimen to eradicate *H. pylori*: rabeprazole capsules (10 mg bid), amoxicillin (1 g bid), clarithromycin (0.5 g bid), and pectin bismuth (300 mg bid), for 14 days. If a family member was confirmed to be infected as well, the same treatment was given to the family member. One month after the end of treatment, the subjects who were *H. pylori* negative, as confirmed by the ^14^C-urea breath test, were included in this study. The eradication was confirmed according to the Fifth National Consensus Opinion on the Diagnosis and Treatment of *H. pylori* [6].

The inclusion criteria were as follows: age between 18 and 65 years; not a recurrent patient; no use of proton pump inhibitors, H2-receptor antagonists, expectorants, or antibiotics within 4 weeks; no related drug allergy history; no gastrointestinal bleeding, pyloric obstruction, perforation, or other complications; no history of digestive tract surgery; and no serious heart, lung, liver, or kidney dysfunction. Exclusion criteria were patients with severe gastric epithelial dysplasia, a pathological diagnosis of malignancy, or lactating or pregnant women.

The protocol of this prospective study was approved by the Ethics Committee of Jiangjin Central Hospital of Chongqing. All patients with *H. pylori* infection, as confirmed by the ^14^C-urea breath test, were enrolled after informed consent.

### 2.2. Data Collection

Gender, smoking history, drinking history, and frequency of eating out (seldom, <1 per month; sometimes, <1 per week and >1 per month; and often, >1 per week) were recorded. One year later, the recurrence of *H. pylori* infection was measured by the ^14^C-urea breath test.

### 2.3. Statistical Methods

Statistical analysis was performed using the SPSS 17.0 software. Continuous data are expressed as the mean ± standard deviation (SD). The count data are expressed as a percentage. Comparisons between two groups were performed using the *t* test or *χ*^2^ test. Variants with a *P* value < 0.2 in the univariate analysis were included in the multivariate analysis. Multivariate logistic regression analysis was performed to determine the independent risk factors for *H. pylori* infection recurrence. The difference was considered statistically significant at *P* < 0.05.

## 3. Results

### 3.1. Clinical Characteristics of the Participants

**Figure 1:**
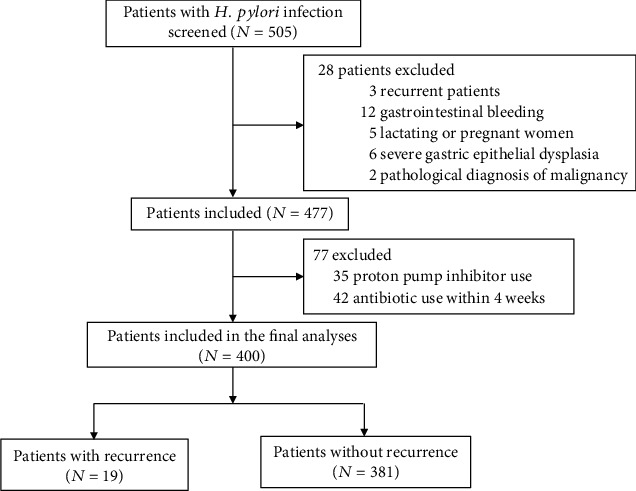
Flow chart of the present study.

The flow chart of the present study is presented in Figure 1, and the baseline characteristics of the participants are shown in Table 1. In total, 400 patients (225 males and 175 females) aged 18–65 years were included. Among them, 19 (4.75%) patients tested positive for *H. pylori* infection at 1 year after eradication. The recurrence rate was 5.33% (12/225) in males and 4.57% (7/175) in females, with no statistical difference (*χ*2 = 0.387, *P* = 0.534).

### 3.2. Relationship between Recurrence Rate and Smoking

Of all 400 *H. pylori*-eradicated patients, 128 were smokers and 272 were nonsmokers. The recurrence rate was 7.03% (9/128) in smokers and 3.68% (10/262) in nonsmokers, with no statistical difference (*χ*2 = 1.947, *P* = 0.163; Table 2).

### 3.3. Relationship between Recurrence Rate and Drinking Alcohol

Of all 400 *H. pylori*-eradicated patients, 186 drank alcohol and 214 did not drink alcohol. The recurrence rate was 6.45% (12/186) in drinkers and 3.27% (7/207) in nondrinkers, with no statistical difference (*χ*2 = 2.019, *P* = 0.155; Table 3).

### 3.4. Relationship between Recurrence Rate and Frequency of Dining Out

Of all 400 *H. pylori*-eradicated patients, 159 patients seldom ate outside the home, 137 sometimes ate out, and 104 often ate out. The recurrence rate was 0.63% (1/159) in the seldom ate out group, 2.92% (4/137) in the sometimes ate out group, and 13.46% (14/104) in the often ate out group (*χ*2 = 13.739, *P* = 0.001; Table 4). The recurrence rate was significantly higher in the often ate out group than in the other two groups.

### 3.5. Relationship between Recurrence Rate and Treatment of Infected Family Members

Of all *H. pylori*-eradicated patients, 104 had *H. pylori*-infected family members who were treated and 296 did not have infected family members or the infection status of their family members was unknown. The recurrence rate was 0.96% (1/104) in those with *H. pylori*-infected family members who were treated and 6.08% (18/296) in those without *H. pylori*-infected family members or the infection status of their family members was unknown, showing a significant difference (*χ*2 = 4.458, *P* = 0.035; Table 5).

### 3.6. Drinking and Dining Out Were Independent Risk Factors for *H. pylori* Infection Recurrence

Next, multivariate logistic regression analysis was performed to determine the independent risk factors for *H. pylori* infection recurrence. The results showed that drinking and dining out were independent risk factors for *H. pylori* infection recurrence (*P* = 0.014 for drinkers and *P* = 0.015 and *P* = 0.003 for those who sometimes and often dine out, respectively; Table 6).

## 4. Discussion

The present study investigated the recurrence rate of *H. pylori* infection after eradication in Jiangjin District, Chongqing, China. Our data showed that the recurrence rate was 4.75%. There was no statistical difference in terms of gender, smoking history, or drinking history, while there were statistical differences between those who frequently ate out compared to those who did not as well as between those whose family members were treated and those who did not have treated family members. Multivariate logistic regression showed that drinking and dining out were independent risk factors for *H. pylori* infection recurrence.

There are two ways that *H. pylori* infection reoccurs after eradication in the stomach [20]. One is recrudescence, which is caused by possible residual strains that multiply, including *H. pylori* that is not completely removed by the treatment. This occurs when the sensitivity of *H. pylori* detection methods is too low, there is a high false-negative rate, or changes occur in the *H. pylori* morphology. Sometimes, the spiral bacteria may transform into spherical bacteria when the environment is poor, and then they return to the spiral form when the environment is suitable, thus causing disease [21]. The other way that *H. pylori* infection reoccurs is reinfection of new strains, including *H. pylori* in oral repositories. These new strains may cause *H. pylori* recurrence when the bacteria move from the mouth into the stomach. Reinfection may also occur due to close contact between people or to exposure to a common source of infection [20].

The recurrence rate of *H. pylori* varies from place to place and is related to the local socioeconomic level [9]. The lower the economic rate, the higher the recurrence rate. For example, the 1-year recurrence rate in Latin America is as high as 11.5%, while the 1-year recurrence rate in Morocco is only 0.45% [18, 22]. The present study found that the recurrence rate of *H. pylori* infection after eradication in Jiangjin District was 4.75%, which is a little higher than the international average (4.3%) [9, 23]. The annual recurrence rate of *H. pylori* infection after eradication is reported to be 1.08–1.75% in China [24, 25]. In addition, a meta-analysis has revealed that the pooled *H. pylori* recurrence rate in China is 2.2% (95% CI: 0.8–4%) [9]. The recurrence rate in the present study was a little higher than that reported previously, which may be caused by many factors, such as location, economic level, scheme selection, drug compliance, and detection method. The latest multicenter prospective study has suggested that ethnic groups, education level, family history, and residence location are independent risk factors for *H. pylori* recurrence [26]. The authors found that the recurrence rate of the residence located in Western China was significantly higher than that of other places, which may partially explain the high recurrence rate of *H. pylori* infection in the present study, since Chongqing is a western city in China. Of note, the recurrence rate for males was slightly higher than that for females, although the difference is not significant.

There are many risk factors for *H. pylori* infection, including socioeconomic factors, education, family density, lifestyle, and other factors [13–16]. Some studies have suggested that smoking and drinking alcohol can reduce the *H. pylori* infection rate [27–29], while other studies show that smoking has no relationship with *H. pylori* infection [30–32]. This study showed that smoking and drinking do not lead to an increase in the recurrence of *H. pylori* infection. However, multivariate logistic regression revealed that drinking was an independent risk factor for *H. pylori* infection recurrence. A study by Amini et al. [33] has demonstrated that the long-term use of common tableware can lead to a high incidence of *H. pylori* infection, indicating that changing dietary habits may limit the spread of *H. pylori* infection. Our study showed that patients who ate out often had a significantly higher recurrence rate of *H. pylori* infection than those who ate out sometimes or rarely. Moreover, multivariate logistic regression indicated that dining out was an independent risk factor for *H. pylori* infection recurrence. This may be because frequent diners have a higher exposure to the source of infection.

*H. pylori* infection has the characteristics of intrafamily transmission. When the infection is successfully eradicated from a patient, he will become reinfected by the same *H. pylori* strain [34] carried by the spouse. Simultaneous treatment of *H. pylori*-infected patients in the same family will significantly improve the eradication rate [35, 36]. This study also confirmed that the recurrence rate in patients whose *H. pylori*-infected relatives living with them also received treatment was significantly lower than that of patients receiving treatment alone. Our results indicate that *H. pylori* infection showed family aggregation, which may be related to close contact, having the same living and eating habits, and exposure to common sources of infection. The cotherapy of family members can improve the therapeutic effect.

## 5. Conclusions

In conclusion, our data showed that the recurrence rate of *H. pylori* infection in Jiangjin District, China, is generally low and not related to gender, smoking, or drinking; however, the recurrence rate is related to the frequency of eating out and the family treatment strategy. Furthermore, drinking and dining out were independent risk factors for *H. pylori* infection recurrence. Our study suggests that the rate of recurrence of *H. pylori* infection may be reduced by limiting the frequency of eating out and receiving cotreatment with family members. Due to the small number of cases included in this study and the short follow-up period, a multicenter, large-scale, randomized controlled trial is needed to confirm our conclusion.

## Figures and Tables

**Table 1:** Clinical characteristics of the participants.

	Total patients (*N* = 400)	Patients with recurrence (*N* = 19)	Patients without recurrence (*N* = 381)	*P* value
Age, mean ± SD	37.8 ± 12.5	39.23 ± 15.3	34.36 ± 14.6	0.653
Sex (male/female)	225/175	12/7	213/168	0.534

**Table 2:** Recurrence of *H. pylori* infection in smokers and nonsmokers.

Group	Number of patients with recurrence	Number of patients without recurrence	Total patients	Recurrence rate
Smokers	9	119	128	7.03%
Nonsmokers	10	262	272	3.68%
*χ* ^2^				1.947
*P*				0.163

**Table 3:** Recurrence of *H. pylori* infection in drinkers and nondrinkers.

Group	Number of patients with recurrence	Number of patients without recurrence	Total patients	Recurrence rate
Drinkers	12	174	186	6.45%
Nondrinkers	7	207	214	3.27%
*χ* ^2^				2.019
*P*				0.155

**Table 4:** Recurrence of *H. pylori* infection in patients with different frequencies of dining out.

Frequency of dining out	Number of patients with recurrence	Number of patients without recurrence	Total patients	Recurrence rate
Seldom	1	158	159	0.63%
Sometimes	4	133	137	2.92%
Often	14	90	104	13.46%
*χ* ^2^				13.739
*P*				0.001

**Table 5:** Effect of the treatment strategy on the recurrence of *H. pylori* infection.

Treatment strategy	Number of patients with recurrence	Number of patients without recurrence	Total patients	Recurrence rate
Treatment alone	18	278	296	6.08%
Cotherapy	1	103	104	0.96%
*χ* ^2^				4.458
*P*				0.035

**Table 6:** Multivariate analysis for independent risk factors for *H. pylori* infection recurrence.

Variants	*P* value	OR	95% CI	
			Upper	Lower
Smoker				
No	Reference			
Yes	0.086	2.425	0.882	6.666
Drinker				
No	Reference			
Yes	0.014	3.711	1.303	10.568
Frequency of dining out				
Seldom	Reference			
Sometimes	0.015	35.665	1.981	642.005
Often	0.003	6.45	1.926	21.6
Treatment strategy				
Alone	Reference			
Cotherapy	0.861	1.298	0.069	24.381

OR: odds ratio; CI: confidence interval.

## Data Availability

The datasets generated and analyzed during the present study are available from the corresponding author on reasonable request.
